# Spatial distribution and male mating success of *Anopheles gambiae *swarms

**DOI:** 10.1186/1471-2148-11-184

**Published:** 2011-06-28

**Authors:** Abdoulaye Diabaté, Alpha S Yaro, Adama Dao, Moussa Diallo, Diana L Huestis, Tovi Lehmann

**Affiliations:** 1 Laboratory of Malaria and Vector Research, National Institute of Allergy and Infectious Diseases, National Institutes of Health, Rockville, Maryland 20852, USA; 2 Institut de Recherche en Sciences de la Santé/Centre Muraz, Bobo-Dioulasso, Burkina Faso; 3 Malaria Research and Training Center, University of Bamako, Bamako, Mali

**Keywords:** Anopheles gambiae, mating success, lek, scramble competition

## Abstract

**Background:**

*Anopheles gambiae *mates in flight at particular mating sites over specific landmarks known as swarm markers. The swarms are composed of males; females typically approach a swarm, and leave *in copula*. This mating aggregation looks like a lek, but appears to lack the component of female choice. To investigate the possible mechanisms promoting the evolution of swarming in this mosquito species, we looked at the variation in mating success between swarms and discussed the factors that structure it in light of the three major lekking models, known as the female preference model, the hotspot model, and the hotshot model.

**Results:**

We found substantial variation in swarm size and in mating success between swarms. A strong correlation between swarm size and mating success was observed, and consistent with the hotspot model of lek formation, the *per capita *mating success of individual males did not increase with swarm size. For the spatial distribution of swarms, our results revealed that some display sites were more attractive to both males and females and that females were more attracted to large swarms. While the swarm markers we recognize help us in localizing swarms, they did not account for the variation in swarm size or in the swarm mating success, suggesting that mosquitoes probably are attracted to these markers, but also perceive and respond to other aspects of the swarming site.

**Conclusions:**

Characterizing the mating system of a species helps understand how this species has evolved and how selective pressures operate on male and female traits. The current study looked at male mating success of *An. gambiae *and discussed possible factors that account for its variation. We found that swarms of *An. gambiae *conform to the hotspot model of lek formation. But because swarms may lack the female choice component, we propose that the *An. gambiae *mating system is a lek-like system that incorporates characteristics pertaining to other mating systems such as scramble mating competition.

## Background

Lekking behaviour is a frequent and conspicuous type of mating aggregation where males gather and display to prospective mates [1], and references therein. Display territories do not hold resources attractive to females other than the males themselves, hence it is assumed that females visit leks solely to copulate [2]. Lekking mating systems are characterized by (i) male clustering; (ii) no male parental care; (iii) no resource on the territory; (iv) fighting over male territories; (v) females mate choice; and (vi) in many cases, stability of lek location over time [3], and references therein. Although the lek mating system has stimulated much interest over the years, it is still not clear why males of some species aggregate during the mating season to attract females. The forces driving the formation of such aggregations have yet to be defined. Further, leks are typically characterized by an unusually high skew in male mating success [4]. Whether female choice is the only reason, or if it acts in concert with, or in opposition to, male competition, is a critical issue for understanding lekking behaviour [5].

In comparison with other territorial mating systems, the extreme clustering of lekking male territories raises the ultimate question of the benefits of this aggregative behaviour in contrast to its associated cost such as competition [6,7]. Why would certain males consent to join others in a mating arena if they do not have any chance to increase their own mating prospect? Three major models have been put forward to explain this paradox. First, lekking behaviour may have evolved as a result of female preference. This model proposes that females prefer to mate with males participating in aggregations either because such aggregations facilitate comparison of several males at a low search cost, or because they offer sites safe from predators. This model assumes that the *per capita *male mating success will increase with lek size [8,9]. Second, the 'hotspot hypothesis' suggests that certain sites are particularly attractive to females (e.g., resource patches) and that males tend to aggregate near these places to intercept the largest numbers of receptive females [10]. The locations of leks are determined by the overlapping female home ranges, mostly around the locations of resources. This model predicts that both male and female numbers will be positively correlated and thus larger leks will have more females, while the *per capita *male mating success remains constant [11,12]. Lastly, the 'hotshot' or attractiveness hypothesis predicts that leks form as a result of high variance in male mating success, such that males with low mating prospects clustering around those that are successful [13]. Lek structure and placement under this model are driven by male-male interactions and one would expect strong phenotypic variation among males across swarms. These three models have been tested in different lek mating systems with various outcomes, but mosquito mating systems have been overlooked.

*Anopheles gambiae*, the major malaria vector in Africa, mates in flight at specific mating sites over landmarks known as swarm markers [14-18]. Swarms are composed of males with females typically entering a swarm and leaving *in copula*. The mating stations contain no resources and females visit these stations solely to copulate. While this mating system is characterized by no parental care, it is not clear whether or not females exhibit mate choice, nor is it clear if males defend their territories. Mate choice is characterized by differences in female responses to possible mates; thus mate choice requires discrimination. In *An. gambiae*, it is thought that the process of copulation is so quick that a female cannot select who to mate with [15,19], hence the mating system of *An. gambiae *does not neatly fall in the lekking category. However given the similarities with the lekking behaviour, we have drawn parallels in order to better understand this mosquito species's mating system.

Characterizing the mating system of a species helps understand how this species has evolved and how selective pressures operate on male and female traits [20]. In the current study, we looked at the variation in mating success between swarms and its underlying factors. How do the mating patterns influence swarm size and placement? Specifically, we tested whether females prefer to mate at large swarms, and whether the *per capita *male mating success varies between swarms. Given that males feed only on sugar but females feed on blood and to some extent on sugar, we tested whether swarms will be clustered around human habitations to increase their encounter rate with females. And finally, we looked at male body-size variation between swarms as a result of mating variation across swarms. Our results are discussed in the context of lekking and others mating systems.

## Results

### Swarm characteristics

**Figure 1:**
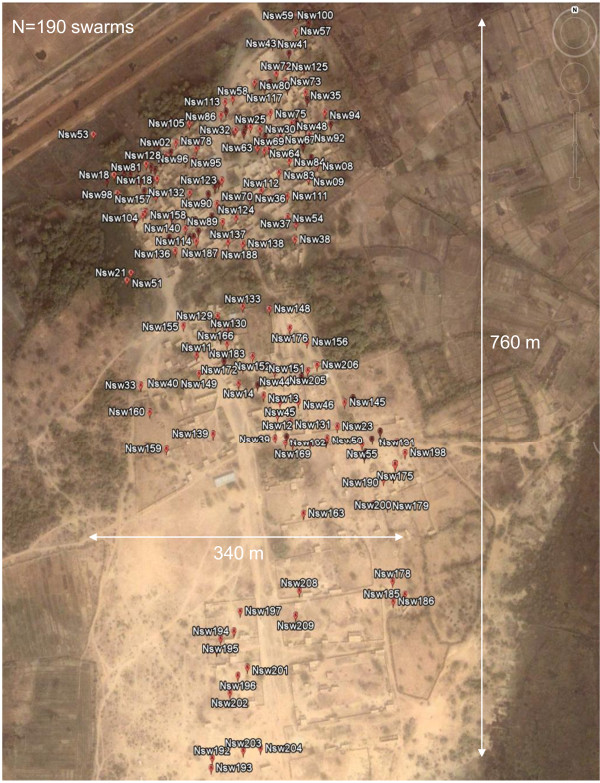
**The map of all swarms (N = 190) found during the survey overlaid on a satellite map (Google-Earth)**. The full area bounded by the road (top left) and the swampy flood zone (bottom right) was included in the survey. The maximal North-South and East-West distances between the most extreme swarms are shown by the white arrows.

**Figure 2:**
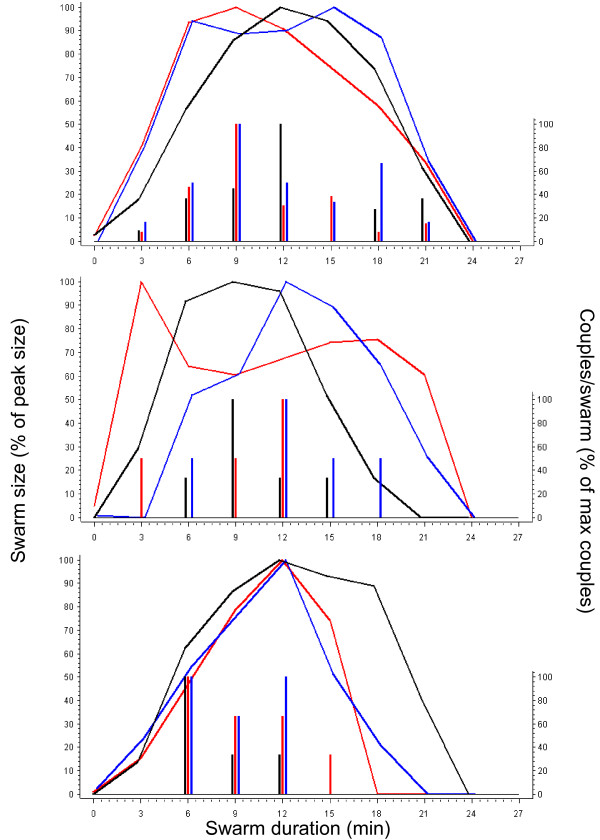
**Swarm size dynamics (line graphs) over time in relation to mating dynamics (vertical bars)**. The nine swarms were observed over one evening (18 August 2008) and divided arbitrarily into three panels to avoid clutter. Each color in each panel represents a distinct swarm measured from the first sighting of swarming in that evening until the last male disappeared. To accommodate variation between swarms in absolute size, the number of males in each swarm and time point was expressed as the fraction of the peak swarm size. Likewise the number of couples per interval of 3 minutes is presented as the fraction from the corresponding maximal value of that swarm. Note that the scale of the couples was compressed and values were jittered horizontally by up to ± 0.3 minute to avoid clutter.

During the survey, a total of 190 swarms were observed, sampled, and mapped across the focal village (Figure 1). Most swarms were species-specific, exclusively composed of *An. gambiae *(97.4%), and the mixed swarms were composed of a mix of *An. gambiae *and one other species, including *An. rufipes, An. pharoensis*, and *Culex *spp. In all mixed swarms, *An. gambiae *was the dominant species (~95%). Genotyping of 30 *An. gambiae *s.l. specimens from each swarm revealed that all were of the M molecular form. Swarms were typically observed 1-3 m above ground at the same site every evening, so observers could wait in advance of the first male's arrival to the site and monitor changes in swarm size. Swarming started approximately five minutes after sunset by one or two males flying in the characteristic zigzag flight. Their numbers rapidly increased within five minutes and reached their peak in 9-15 min; then slowly decreased to nearly zero 25-30 min after the first male(s) was seen (Figure 2). Couples were usually observed from six minutes after the first males started to swarm and continued for the next 10 minutes, during which time the swarm was near its peak size (Figure 2).

### Variation in swarm size

**Figure 3:**
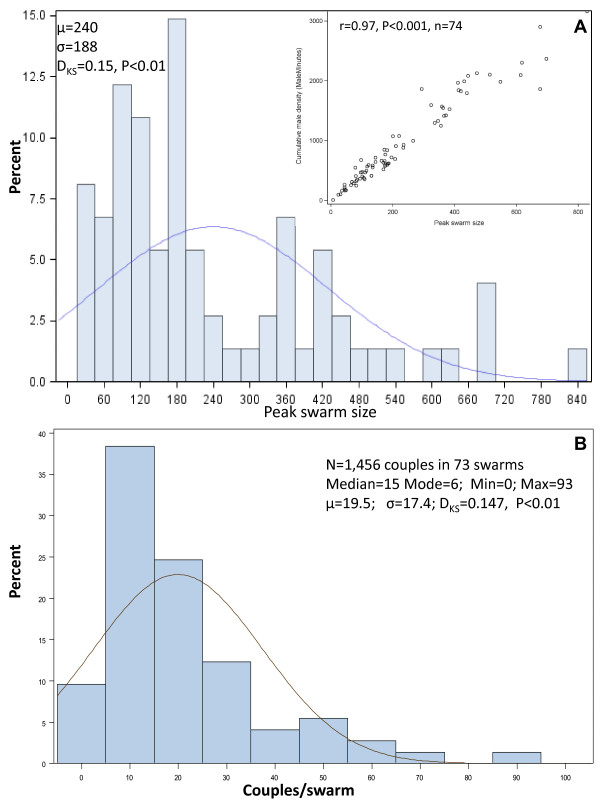
**Distributions of peak swarm size (A) and the total number of couples per swarm (B)**. Normal density curves are depicted based on the data means and standard deviations. The results of the Kolmogorov-Smirnov (KS) tests are reported under the mean and standard deviation. Panel A inset: Relationship between peak swarm size and cumulative swarm size (excluding swarms which were not observed throughout).

Of the 190 swarms that were mapped and characterized during the survey, 74 swarms were observed and photographed every three minutes from formation to the departure of the last male (see Methods). Peak swarm size and the cumulative male numbers (the sum of all males observed every 3 minutes throughout the duration of the swarm) were highly correlated (Figure 3A inset; r = 0.97, P < 0.001, N = 74). This correlation further indicates that the dynamics of swarm size over time were similar among swarms as described in Figure 2. Specifically, this correlation suggests that there were no short-duration, high-density swarms or long-duration, low-density swarms. Henceforth, we used peak swarm size as our overall measure of swarm size. The distribution of peak swarm size did not fit a normal distribution (Figure 3A; P < 0.01, Kolmogorov-Smirnov [KS] Test) and suggested multiple modes. Specifically, the distribution's main modes were near 100 and 180 males, with secondary modes near 360 and 420, and possibly another mode near 690 (Figure 3A). A multimodal distribution suggests that the corresponding swarm sizes maximize male mating success, but it may also reflect variation between marker types, dates, or areas of high and low swarming activity (zone).

**Figure 4:**
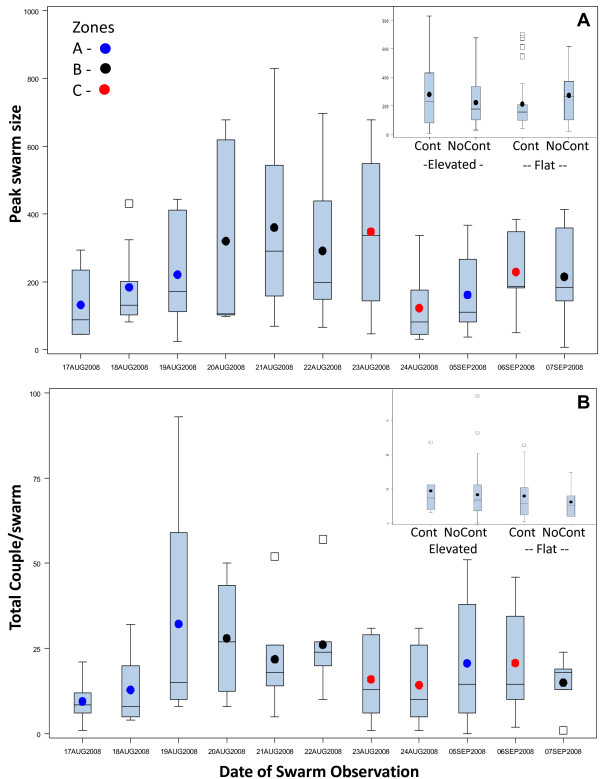
**Variation in peak swarm size (A) and the total number of couples per swarm (B) over time in each of the three zones**. The box of each box whisker plot extends from the first to the third quartiles (the interquartile range, IQR), the whiskers extend to extreme observations up to 1.5 times the IQR, beyond which observations are marked by squares. The median and the mean are marked by line and a dot. The color of the dot indicates the zone. Sample size per date ranged between 7 and 10 swarms. Insets: Variation in swarm size (A) and the total number of couples per swarm (B) among marker types.

Accordingly, the effect of the marker type was evaluated in an ANOVA model accounting for the effects of date and the swarming site as random effects (Figure 4A, Table 1). The effects of marker type and date were insignificant, whereas swarming site was highly significant (Table 1) and accounted for 47.6% of the total variance in swarm size, suggesting that mosquitoes respond to other marker properties. The distribution of the residuals was unimodal and nearly symmetrical, although it departed from normality (KS Test, D = 0.16, P < 0.01, not shown). The log-transformed data did not depart from normality (KS Test, D = 0.09, P < 0.1, not shown). The effect of zone was also insignificant (Figure 4A, Table 2). These results indicated that overall, swarming activity was stable (over a period of two weeks), and that the differences in swarm size between sites remained stable over this period, suggesting that the variation in swarm size is governed by site-specific, as-yet unidentified, factors.

**Table 1:** ANOVA results showing the effect of marker type (fixed), zone (random), and date (random) on peak swarm size and on total couples/swarm

	Peak swarm size		Total couples/swarm	
**Variable**	**F/Z^a^**	**P**	**F/Z**	**P**

Marker Type	0.40	0.75	0.26	0.85
Site	2.70	0.003	2.35	0.009
Date	1.02	0.155	1.41	0.079
Residual	4.38	0.001	4.80	0.001

Res -2LL	963.5		573.2	
AIC	969.5		579.2	

The degrees of freedom for the F-test of "Marker Type" were 3 for the numerator and 37, and 33, for the denominator of swarm size and total couples, respectively (four swarms with size estimates had no complete measurement of total couples).

^a^F denotes that the standard F-test for fixed effects and Z denotes Wald-Z tests for the covariance parameter estimates

**Table 2:** ANOVA results testing the random effects of zone, swarm-site, and date on peak swarm size and on total couples/swarm

	Peak swarm size		Total couples/swarm	
**Variable**	**Z^a^**	**P**	**Z**	**P**

Zone	0.15	0.44	.	-
Site(Zone)	2.7	0.004	2.36	0.009
Date(Zone)	0.93	0.17	1.30	0.098
Residual	4.4	0.001	4.21	0.001

Res -2LL	1022.1		608.8	
AIC	1030.1		614.8	

^a ^Z denotes Wald-Z tests for the covariance parameter estimates

### Variation in mating success between swarms

Overall, 1,456 mating couples were observed in 70 swarms (located at 30 swarming sites) over 11 days. The number of mating events per swarm varied from zero to 93, with a mode of six and a possible secondary mode around 50 (Figure 3B). This distribution did not follow normal expectations (Figure 3, KS Test, D = 0.15, P < 0.01) and exhibited substantial variation, suggesting heterogeneity in swarm mating success (Figure 3B). To evaluate the effects of the marker type, zone, and date on the number of couples per swarm, we performed similar analyses as described above for swarm size (Figure 4, Tables 1 and 2). As in the case of swarm size, only swarming site had a significant effect on the number of couples per swarm and it alone accounted for 40% of the total variance in swarm mating success, whereas marker type, zone, and date had no significant effect (Figure 4B, Tables 1 and 2).

**Figure 5:**
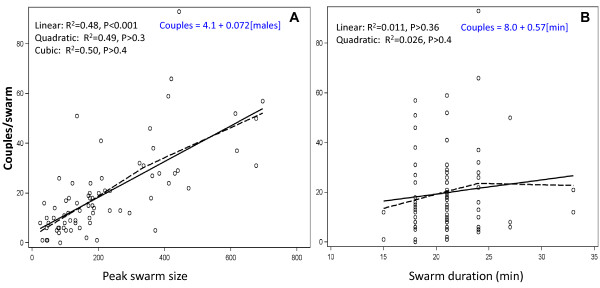
**The effect of swarm size (A) and duration (B) on swarm mating success**. Simple linear regression (solid line) and locally-weighted scatterplot smoothing (LOESS, broken line) are used to evaluate the trends. Overall model statistics for the linear and polynomial regression models are shown (see text for details). Equations of the linear models are shown in blue.

In order to evaluate the effect of swarming duration and swarm size on mating success, we used univariate and multivariate analyses. Mating success was significantly and positively related to swarm size, as indicated by the linear, univariate model (Figure 5A). The Loess curve suggested a point of curvature, which was tested for with quadratic and cubic models, but only the linear effect was significant (Figure 5A, P > 0.3 for both the quadratic and the cubic effects, P < 0.001 for the linear effect). A lack-of-fit test (after rounding the number of males to the nearest 10) further confirmed that the effect of swarm size is linear (P > 0.7 for the nonlinear component). However, despite a trend showing an increase in mating success with swarming duration from 15 to 25 minutes (Figure 5B, Loess curve), the effect of swarm duration on mating success was not significant when tested as a linear or quadratic model (Figure 5B). Likewise, swarm duration (and its quadratic term) was not significant in the final model that includes all explanatory variables (P > 0.18, Table 3 see below).

**Table 3:** ANOVA results showing the effect of marker type, swarm size, swarm duration, and the square of swarm duration, as well as the random effects of date and swarming site on mating success (total couples/swarm)

Variable	Estimate	F/Z^a^	DF (num/den)^b^	P
Marker	--	1.22	3/30	0.31
Duration	5.5	2.52	1/30	0.12
Duration^2^	-0.11	2.68	1/30	0.11
Swarm Size	0.072	57.9	1/30	0.001
Site	26.16	1.23	--	0.109
Date	28.15	1.33	--	0.092
Residual	109.95	4.47	--	0.001

Res -2LL	542.4			
AIC	548.4			
				

^a^F denotes the standard F-test for fixed effects and Z denotes Wald-Z tests for the covariance parameter estimates

^b^Degrees of freedom of the numerator (num) and denominator (den) of the F-tests

In the multivariate analysis, swarm size was the only significant factor, with swarm site no longer being significant (Table 3), suggesting that swarm size better predicted swarm mating success than swarming site, although site was the best predictor of swarm size (above). These positive relationships suggest that both males and females are more attracted to certain display sites, but that females are further attracted to larger swarms, or that daily conditions such as wind and clouds modulate the specific attraction of the site. Approximately 7% of the males in a swarm will mate every night (estimate = 0.072, SE = 0.0095; Table 3), assuming that males mate only once per evening. So a limitation of male numbers cannot drive this relationship. The slope (0.072) reflects an additional mating event with every 14 additional males in the swarm. Most importantly, the linear effect of swarm size on its mating success reveals that individual male success is similar regardless if he joins a large or small swarm.

**Figure 6:**
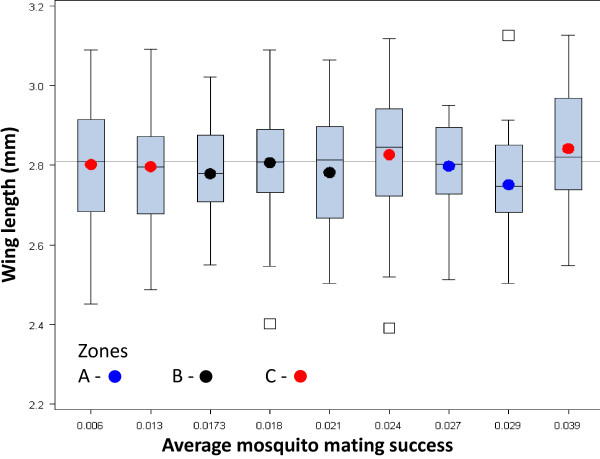
**Variation in male body size (wing length) among ten swarms**. Swarms are ordered according to the per-capita mating probability. The mean wing length of each swarm is marked by a dot, and its color indicates the zone. The box whisker plots are explained in Figure 4. Overall mean body size (2.81 mm) is indicated by the horizontal line. Sample size per swarm ranged between 21 and 95.

The between-swarm variation in male body size (N_swarms _= 10, N_males _= 491) was examined in relation to the random effects of date and swarm (within date) as well as the fixed effect of swarm "phase" (early, middle, or late; see Methods). None of these factors were significant (P > 0.26; data not shown). Virtually the same results were obtained when zone replaced date (data not shown). Finding no evidence for variation in body size between males of different swarms, no further testing was performed on the effect of male size on swarm mating success. However, no such relationship is apparent upon visual examination (Figure 6).

### Spatial variation in swarm mating success

**Figure 7:**
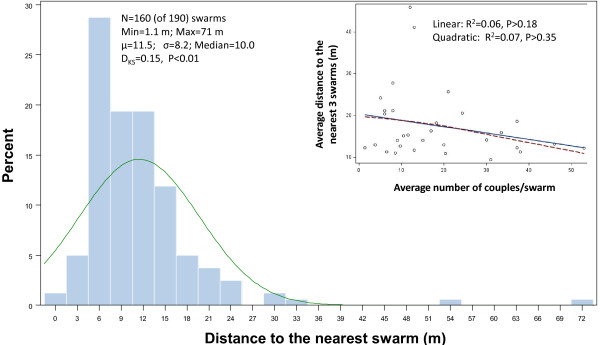
**Distribution of distance to the nearest swarm**. To minimize the edge effect on the distribution, distances were computed for all swarms, but only those included in an inner diameter (N = 160) were included in the distribution shown above. Inset: the effect of overall swarm mating success on the average distance to the nearest 3 swarms to test that swarms cluster near highly successful swarms. Simple regression (solid line) and LOESS (broken line) are used to evaluate the trends. Quadratic and linear regression summary statistics are shown.

Despite extensive efforts to locate swarms throughout the predefined area, the swarms (N = 190) were concentrated in a central stretch along the village residential area with a higher density in the northern part of the village (Figure 1). The median distance between any swarm and the nearest swarm was 10 m, and the distance to the nearest swarm of 25% of the swarms was smaller than 6.5 m (Figure 7), suggesting that interactions between swarms are possible. To determine if swarms clustered near other swarms with high mating success, we computed the average distance of each swarm with its 3 nearest neighbours and regressed this average over the total number of couples per swarm. Because most swarms were observed over several evenings and the effect of date on mating success was not significant (Tables 1, 2 and 3), overall swarm mating success was computed as the average number of couples over the nights the swarm was observed. We expected the distance to the nearest three neighbouring swarms to decrease with increasing mating success. The regression slope was negative, but insignificant (P > 0.18, Figure 7). Likewise, the quadratic polynomial regression was insignificant (P > 0.35, Figure 7). These results do not support clustering of swarms around highly successful swarms.

## Discussion

The role of male-male competition and female choice in shaping the mating systems in many lek-breeding animals is well-known but has been overlooked in mosquitoes. For example, little is known about the forces that constrain swarm spatial distributions and modulate the variation in mating success between and within swarms. Our main goals in the present study were to describe the variation in mating success between swarms and explore the factors that structure it. We expected to find large variation in swarm mating success between swarms and we presumed that this variation would reflect the difference in mating success of the males in different swarms. Surprisingly, our results reveal that despite substantial variation in mating success between swarms, the prospects of individual males to mate are independent from the swarm they are in. These results suggest that males may not be able to increase their individual mating success (fitness) by swarm selection. Consistent with this conclusion, we find no evidence that swarms cluster around a highly successful swarm, as there was no significant reduction in the average distance to the nearest three swarms with increasing mating success of a swarm. Further, significant variation in swarm size was found across space, and swarm site was the major factor that accounted for this variation. How then do these observations relate to the predictions of the different models for the evolution of leks?

Several different selective forces may have led to lekking behavior. The two major forces in play are male competition and female choice [3,6,10]. If leks are male-initiated, intense male competition might be an important evolutionary force. The dispersion pattern of males within leks should reflect the males' interests and might constrain the future evolution of female choice. If, however, leks are female-initiated, males should be distributed in ways that allow for effective choice [2,21]. Of the different models of lek formation, the female preference model is the only one that posits that female choice is the driving force [3,6,10]. Females prefer to find a mate among a group of males because such aggregations allow for greater and easier discrimination of potential mates [9,22]. Consequently, males would be selected to lek because females preferentially mate in large aggregations [23]. In such a scenario, we would expect females to discriminate among swarms. The total number of females and the *per capita *mating success of an individual male should increase with swarm size [12,24]. While we found a strong correlation between the number of mating events and swarm size, male mating success did not increase with increasing aggregation size; hence our results are inconsistent with the female preference model.

The hotspot model of lek formation assumes that leks are male-initiated [10]. Males set themselves in places where the probability of encountering receptive females is the highest. Males have no direct information about female location, but they use cues from non-defendable resources that females use to set their sites. Consequently, the highest density of males will coincide with the highest density of females and one would then expect strong variation in swarm size and in mating success between swarms across space, but the individual mating success of males should be the same across space [11,12]. Our results are consistent with this view, and hence are in agreement with the hotspot model. Several insect mating systems conform to the hotspot model including the medfly [25], sandfly [26] and fruit fly [27]. Niyazi and Shuker [25] showed that females of *Ceratitis capitata *are attracted to preferred sites, which are characterized by their exposure to sunlight. In a study on lekking behaviour of a Hawaiian *Drosophila*, Droney [27] found that males initiate leks at locations in proximity to food/oviposition substrates to maximize their chance of encountering females. In the current study, swarms of *An. gambiae *were not randomly distributed over space. Rather, a spatial clustering was observed. A visual inspection of swarms' spatial structure showed an area of high and low swarm density. The area of high density perfectly matched the spatial distribution of human habitations, consistent with the data collected by Charlwood et al. [28] in Mozambique. In their study, swarms of *An. funestus *were mostly found close to human habitations where virgin females rest [28]. Our results on swarm spatial distribution thus conform to the hotspot predictions.

The hotshot model of lek formation is male-initiated and argues that "poor quality" males gather around successful males to parasitize their greater attractiveness [29, and references therein]. Our data do not allow direct interpretations of the intrinsic mating success of individual males within swarms. However as suggested by a few authors [15,19], if females are instantly seized and inseminated as soon as they enter the swarms, the strategic position of males within a swarm and the flight performance of males should determine their mating success as shown in *Chironomus plumosus *[30]. Successful "maters" in this swarming insect are thought to have better aerobatic ability. Large body size is another trait known to be correlated with male mating success, but this correlation has not been found in *An. gambiae *[31]. Our results did not show any evidence for variation in body size between males of different swarms. In contrast, Yuval et al. [32] showed that swarming males of *An. freeborni *were larger than non-swarming males. In a laboratory experiment, N'ghabi et al. found that intermediate-sized *An. gambiae *males were highly successful in getting mates as opposed to the smallest or largest males [33,34]. While these discrepancies are probably due to variation in mosquito species mating characteristics and/or to ecological differences, they stress the need to further investigate the role of body size in *An. gambiae *male-mating success. Other studies have suggested that male age [35,36], genetics [37], or the sperm length [38] could influence the mating success of a male. Additionally, pheromone release and acoustics may account for mating success of both individual males and swarms [39]. Recent laboratory studies showed that female and male mosquitoes modulate their flight tone harmonics to match that of their mate [40-42]. Finally, females may visually assess swarm size.

Leks may also form and evolve for reasons other that sexual ones. Yuval and Boukila [43] observed that mating activities in *An. freeborni *coincide with predator activity. Although we did not look at predation, we acknowledge that males may aggregate to reduce predation on themselves while swarming and females might choose to mate in large swarms for the same reason.

The mating systems of several insects are lek-based [25-28], but *An. gambiae *fails to fall neatly into that category. While female choice of a sexual partner is an essential characteristic of leks, females of *An. gambiae *may not have time to select among males within the swarm [15,19]. Further, there are no reports of males fighting over territories, another characteristic of lekking behavior. We propose that the *An. gambiae *mating system can thus be considered a lek-like strategy that incorporates characteristics pertaining to other mating systems such as scramble mating competition. This mating system is characterized by a lack of overt conflict. Males outpace each other in the search for suitable mates and males capable of persistent searching are more likely to intercept females than less-active individuals [44]. Females apparently do not exert mate choice in a classical way, since they appear to accept any incoming males. However, since males have to outpace each other in searching for females, only a set of competing males will mate with them [45]. In *An. gambiae*, males which most-actively search for mates in the hyperspace of swarms probably gain the benefit of finding more females.

## Conclusions

The present study reports the swarming and mating behavior of *An. gambiae*. We found large variations in swarm size and mating success between swarms. A strong correlation between swarm size and mating events was observed, but the *per capita *mating success of an individual male was the same across swarms. Although the mating system of *An. gambiae *could not be neatly qualified as lekking, our data are in agreement with the hotspot model of lek formation. We propose that the mating system of *An. gambiae *falls in between a lek and scramble competition. Our data do not allow inferring if swarms follow the hotshot model of lek formation, in which only the highest-quality males get the benefit of the mate-seeking females. Further studies are needed to clarify the mating success of individual males with respect to their quality. Due to the spread and increasing levels of resistance to insecticide, alternative measures based on genetic approaches to controlling malaria transmission are being planned. Knowledge of mosquito mating systems can help assist the implementation of these genetic strategies as well as designing innovative tools.

## Methods

### Study area

Our study on *An. gambiae *swarming behaviour was conducted in the village Sokourani (14°17'N, 8°5'W) in the district of Niono, 350 km northeast of Bamako, Mali. The area has one of the largest rice irrigation systems in West Africa. This rice cultivation area was developed in 1945 [46]. Located in the Sahel, the mean annual rainfall varies between 600 to 800 mm, which falls between June and September. Except for sporadic rains in March that typically do not provide enough surface water for complete development of mosquito larvae, the Sahelian dry season extends from October to May [47]. The river Fala is a permanent source of irrigation for two cycles of rice growing each year (July-November and January-May). The irrigation system and the rice fields present conditions typical of permanent mosquito larval sites. During the wet season, additional temporary larval sites abound. The village has about 600 inhabitants, mainly farmers, with sheep, goats, and a few cows also present. The study was carried out from August to September 2008.

### Swarm survey

A swarm is defined as a collection of mosquitoes that fly in a cohesive stationary cloud, usually less than 1 m radius, after sunset on a given day. Swarms were surveyed at sunset by 30 trained observers across the entire village for 10 days. Every day, another sector of the village was covered. Swarms were sampled using an insect net, and individual mosquitoes were killed with chloroform, identified visually, and kept in 80% ethanol in 1.5-ml tubes. The location of the swarm, the time of collection, the landmarks under the swarm, and the height from the bottom of the swarm to the ground were recorded. Landmarks were classifieds by two criteria: the presence of contrasting light/dark surface (e.g., wells, grasses-footpath intersections) and projections (elevation) above ground (e.g., walls, woodpiles). This generated four classes of marker types, 'Flat-Contrast', 'Elevated-Contrast', 'Flat-No-contrast' and Elevated-No-contrast.' Observations were made on 190 swarming sites spread throughout the village. Swarm locations, households (compounds), and larval sites were mapped using a global positioning system (GPS) with measurements of latitude and longitude accurate to within 2 m. Collected specimens were identified by PCR to species and molecular form [48], and a subset was used to estimate body size. Specifically, males from 10 different swarms were collected repeatedly from the swarm (approximately every five minutes). One wing of each male was removed, mounted, and measured (the distance between the alular notch and the intersection of the radius 3 vein and the outer margin) as previously described [49].

### Swarm size and mating success

After completion of the swarm survey (above) and the mapping of swarms throughout the entire village, three groups of ten swarms each in three different zones were selected for intensive observations. The zones were selected in high and low swarm-density areas. We started by randomly selecting one swarm in each zone, then the nearest nine swarms were subsequently selected. Each group of 10 swarms was surveyed for at least three days. The number of males in a swarm was determined at three-minute intervals from the beginning to the end of the swarm. Digital cameras were used to take pictures of the swarms and these pictures were subsequently used to estimate (by counting) the number of males in each swarm [32]. At the same time, the number of mating events in each swarm was also recorded by an observer using a tally counter. Pairs *in copula *are easily recognized since they have a characteristic flight pattern and they tend to fall out of or fly away from the swarm [14]. We acknowledge that there are some limitations to our measurement of male reproductive success. Pairs *in copula *dropping out of the swarms were counted, however some of these pairs may not result in successful mating.

## Data Analysis

Statistical analyses were performed using SAS 9.2 (SAS Institute, Cary, NC). Goodness-of-fit tests with the normal distribution were performed using Kolmogorov-Smirnov (KS) tests. Certain analyses included fixed (e.g., marker type) and random variables (e.g., swarming site, date, and zone). If random effects were included, mixed models ANOVAs were implemented using PROC MIXED within SAS. Swarm sites and date were nested in zone, because only the swarms of a single zone could be observed each evening. Additionally, our classification of markers based on contrast and height above ground resulted in four categories (above), some of which were not found in all zones. Therefore, we evaluated the effect of zone and marker in separate models. The component of variance attributed to particular categorical (non-continuous) variables such as 'swarm site' was used as an estimate of its "effect" on the response variable. As for repeatability, it was computed as the relative contribution of the factor of interest to the total variance in the response variable using estimates of variance that were derived by PROC MIXED in SAS. This measure of effect size was calculated only for statistically significant categorical factors because the slope coefficient of continuous factors (e.g., swarm size) provides direct measure of the effect size of such factors on the response. To assess if effects were nonlinear, we used (i) locally weighted scatterplot smoothing (LOESS) side-by-side with regression analyses, (ii) polynomial regression according to the curvature suggested by the data and the LOESS, and (iii) lack-of-fit tests to determine if the non-linear component of the variance was significant. To minimize the edge effect in estimating the distance to the nearest swarm(s), distances were computed between all swarms (N = 190), but only swarms located in an inner rectangle (n = 160) were used (Figure 1).

## Competing interests

The authors declare that they have no competing interests.

## Authors' contributions

The work presented here was carried out in collaboration between all authors. ADi and TL designed the study. ADi carried out the field and laboratory work, participated in the analysis of data, interpreted the results, and drafted the paper. ASY, ADa and MD carried out the fieldwork and revised the manuscript. DLH participated in the data analysis and revised the manuscript. TL analysed the data, interpreted the results, and revised the manuscript. All authors have read and approved the final manuscript.
